# Polymyxin B Combined with Minocycline: A Potentially Effective Combination against bla_OXA-23_-harboring CRAB in In Vitro PK/PD Model

**DOI:** 10.3390/molecules27031085

**Published:** 2022-02-06

**Authors:** Xingyi Qu, Xingchen Bian, Yuancheng Chen, Jiali Hu, Xiaolan Huang, Yu Wang, Yaxin Fan, Hailan Wu, Xin Li, Yi Li, Beining Guo, Xiaofen Liu, Jing Zhang

**Affiliations:** 1 Institute of Antibiotics, Huashan Hospital, Fudan University, Shanghai 200040, China; 20111030085@fudan.edu.cn (X.Q.); bxc19940216@163.com (X.B.); hujialifudan@163.com (J.H.); xlanhuang@163.com (X.H.); 13917658241@163.com (Y.W.); fanyaxin20080908@126.com (Y.F.); mswuhl@163.com (H.W.); lxxj6311@163.com (X.L.); 85773627@163.com (Y.L.); 13045666468@163.com (B.G.); 2 Key Laboratory of Clinical Pharmacology of Antibiotics, Shanghai 200040, China; 3 National Health Commission & National Clinical Research Center for Aging and Medicine, Huashan Hospital, Fudan University, Shanghai 200040, China; cyc_1983@163.com; 4 Phase I Unit, Huashan Hospital, Fudan University, Shanghai 200040, China; 5 Department of Biological Medicines & Shanghai Engineering Research Center of Immunotherapeutics, School of Pharmacy, Fudan University, Shanghai 201203, China

**Keywords:** *Acinetobacter baumannii*, polymyxin B, minocycline, combination therapy, in vitro pharmacokinetic/pharmacodynamic model

## Abstract

Polymyxin-based combination therapy is commonly used to treat carbapenem-resistant *Acinetobacter baumannii* (CRAB) infections. In the present study, the bactericidal effect of polymyxin B and minocycline combination was tested in three CRAB strains containing bla_OXA-23_ by the checkerboard assay and in vitro dynamic pharmacokinetics/pharmacodynamics (PK/PD) model. The combination showed synergistic or partial synergistic effect (fractional inhibitory concentration index ≤0.56) on the tested strains in checkboard assays. The antibacterial activity was enhanced in the combination group compared with either monotherapy in in vitro PK/PD model. The combination regimen (simultaneous infusion of 0.75 mg/kg polymyxin B and 100 mg minocycline via 2 h infusion) reduced bacterial colony counts by 0.9–3.5 log_10_ colony forming units per milliliter (CFU/mL) compared with either drug alone at 24 h. In conclusion, 0.75 mg/kg polymyxin B combined with 100 mg minocycline via 2 h infusion could be a promising treatment option for CRAB bloodstream infections.

## 1. Introduction

Bloodstream infections are most common (37/131 studies, 28%) severe infections among reported studies, and *Acinetobacter baumannii* is the top pathogen (52/131 studies, 38.5% and 4395/11,546 patients, 38%) [1]. The mortality of *Acinetobacter baumannii*-related bloodstream infections showed an increasing trend in a 10-year prospective study in China [2]. There is an urgent need for rational and effective medication schemes to be developed in clinics. Polymyxins have become a last-resort treatment for multi-drug resistant Gram-negative bacterial infections, including *Acinetobacter baumannii* [3]. Although higher doses resulted in better therapeutic outcomes, greater incidence of toxicity tended to follow, such as nephrotoxicity and neurotoxicity [4]. The adverse effects limited the clinical use of polymyxins and deficient drug doses may induce the development of polymyxin-resistant Gram-negative bacteria [5]. Polymyxins are concentration-dependent antibiotics [6]. The pharmacokinetics/pharmacodynamics (PK/PD) index of polymyxins is polymyxins’ area under the concentration-time curve (AUC) divided by minimum inhibitory concentration (MIC), which is 7.4–17.6 for 2 log_10_ colony forming units per milliliter (CFU/mL) decrease against *Acinetobacter baumannii* in mouse thigh model [7]. In contrast to colistin methanesulfonate (CMS), whose active drug concentration increases slowly in blood after administration, polymyxin B has remarkable advantages in bloodstream infections [8]. Coupled with the fact that heterogeneous resistance of *Acinetobacter baumannii* occurred when using polymyxin B alone [9,10], polymyxin-based combination therapy is rational and necessary [8].

Minocycline, belonging to tetracyclines, is promising for the treatment of *Acinetobacter baumannii* infections [11,12,13,14]. Minocycline is a time-dependent antibiotic with a long post-antibiotic effect (PAE), the PK/PD index of which is AUC/MIC, and the PK/PD targets for 1 log_10_CFU/mL were 23.3 ± 3.7 against *Acinetobacter baumannii* in in vitro studies [15]. A study initiated by the Antimicrobial Stewardship Program (ASP) found that minocycline was safe and effective for patients infected with multi-drug resistant *Acinetobacter baumannii* (MDRAB) [16]. Several in vitro and in vivo studies have demonstrated synergistic effects and potential mortality decreases of the combination of polymyxin B and minocycline [17,18,19,20]. Polymyxin B and minocycline combination not only had a partial synergy in checkerboard assay with the fractional inhibitory concentration index (FICI) value of 0.75 [17], but also prolonged the survival time of the lung-infection mice [17,18].

However, most previous studies on the combined efficacy of polymyxin B and minocycline against *Acinetobacter baumannii* were limited to checkerboard assays and static time-kill curves [18,19,20], rarely considering the dynamic changes of drugs’ pharmacokinetics in the human body. Therefore, the present study simulated the pharmacokinetics of polymyxin B and minocycline against *Acinetobacter baumannii* bloodstream infection in in vitro dynamic PK/PD models. The bactericidal effects are expected to provide important information for optimizing dosing regimens for this combination against carbapenem-resistant *Acinetobacter baumannii* (CRAB).

## 2. Results

### 2.1. Genetic Information, MICs, and Checkboard Assays

The genetic information of the three strains has been analyzed through whole-genome sequencing [21], as shown in Table 1. The three strains have different multilocus sequence typing (MLST) subtypes, namely, ST195, ST208, and ST191, which were the dominant sequence types in *Acinetobacter baumannii* [21]. All the three strains had the *lpxC* and *lpxA* genes without mutations. The three strains were intermediate to polymyxin B (according to CLSI, M100-S31, 2021) with MICs of 0.5, 0.5, and 0.25 mg/L, respectively (Table 2). All the three strains harboring the *tetA* gene that encodes resistance to tetracycline were resistant to doxycycline (MICs were 64 mg/L for all the strains), susceptible to minocycline for AB070311 and AB170428, and intermediate for AB162487. The genome annotation revealed that all the strains contained multiple antibiotic resistance genes including OXA-23, OXA-66 encoding the beta-lactamase resistance, which are consistent with the MICs over 16 mg/L for meropenem and doripenem. The three strains also had high MICs for aztreonam (MICs = 64 mg/L) and cefoperazone (MICs > 256 mg/L), which were consistent with the ARO gene, *TEM-1*, for example.

Checkboard assays showed that one strain (AB162487) had a synergistic effect of polymyxin B and minocycline with a FICI value of 0.375. The other two strains (AB070311 and AB170428) showed partial synergistic effects of polymyxin B and minocycline, and the FICI values were 0.56 (Table 2).

### 2.2. Dynamic PK/PD Model

#### 2.2.1. Concentration Verification

**Figure 1:**
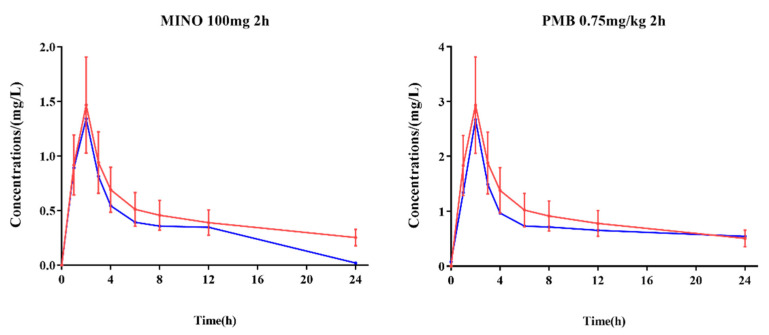
Comparison of target concentrations in human subjects and measured concentrations in the central compartment of in vitro PK/PD model. Red lines: target concentrations in vitro; blue lines: measured concentrations; error limit (red): ±30% of target concentrations.

In the dynamic PK/PD model, the concentrations of polymyxin B (0.75 mg/kg intravenously administration with 2 h infusion) and minocycline (100 mg intravenously administration with 2 h infusion) in the central compartment were within ±30% of the target concentrations (except minocycline at 24 h) as verified by LC-MS/MS (Figure 1).

#### 2.2.2. Time-Kill Curves

**Figure 2:**
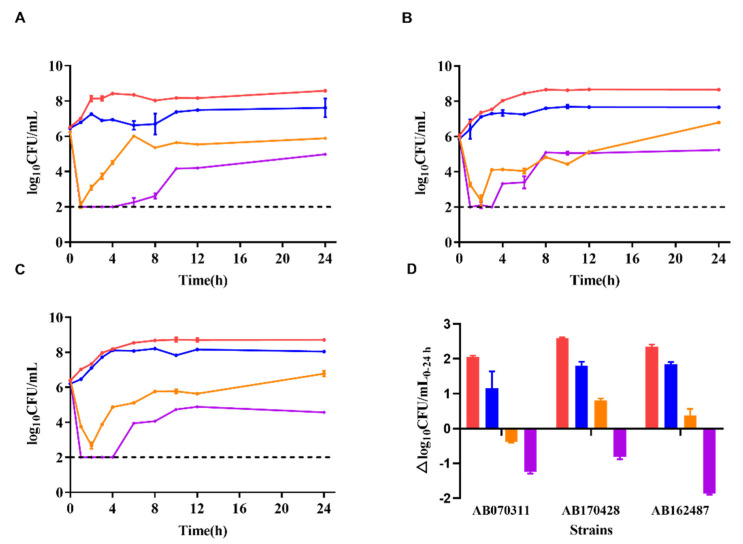
Time-kill curves of polymyxin B and minocycline alone and in combination against CRAB strains in a dynamic PK/PD model. (**A**): Time-kill curves of AB070311, (**B**): time-kill curves of AB170428, (**C**): time-kill curves of AB162487, (**D**): colony counts changed in the range of 0–24 h; red lines or column: bacterial growth curve; blue lines or column: minocycline 100 mg via 2 h infusion; orange lines or column: polymyxin B 0.75 mg/kg via 2 h infusion; purple lines or column: minocycline 100 mg combined polymyxin B 0.75 mg/kg via 2 h infusion; dash line: the detection limit.

The colony counts of the three strains were recorded under different dosing regimens (Figure 2). The single dosing regimen of polymyxin B (pharmacokinetics corresponding to 0.75 mg/kg 2 h infusion in bloodstream infection patients) showed antibacterial activity for the first 2 h, with the colony counts approximately decreased 3–4 log_10_CFU/mL (range from −3.74 to −3.17 log_10_CFU/mL). However, the colony count then increased to the initial inoculum level in the following 22 h. The colony counts of single dosing regimen of minocycline (pharmacokinetics corresponding to 100 mg via 2 h infusion in healthy volunteers) resulted 1.6 log_10_CFU/mL increase in average (ranging from 1.16 to 1.85 log_10_CFU/mL) in 24 h. Nevertheless, the combination regimen of polymyxin B (0.75 mg/kg, 2 h infusion) and minocycline (100 mg, 2 h infusion) demonstrated significantly enhanced antibacterial activity over a 24 h period, with the colony counts decreasing more than 4 log_10_CFU/mL at 3 h (below the limit of detection).

As shown in Figure 2, for the combination therapy, despite a rebound occurring after 3 h, the average colony counts were still decreased by 1.3 log_10_CFU/mL at 24 h compared with initial counts at 0 h (ranging from −1.86 to −0.81 log_10_CFU/mL), showing superior antibacterial activity and 0.9–3.5 log_10_CFU/mL compared with either drug alone at 24 h [22].

#### 2.2.3. PK/PD Calculation

The AUCs were 19.07 and 8.61 mg·h/L for polymyxin B (0.75 mg/kg infusion) and minocycline (100 mg infusion), respectively. The AUC/MICs for polymyxin B and minocycline in monotherapy or combination therapy were calculated in Table 3. In checkboard assays, polymyxin B and minocycline combination decreased MICs by 2–8 folds more than monotherapy did. Therefore, AUC/MIC achieved higher values after combination comparing with that of monotherapy.

## 3. Discussion

Polymyxin-based combination therapies would be effective treatments for CRAB infections, with higher clinical response rates compared with non-polymyxin based therapies [23]. Zhang et al. [20] performed checkerboard assays and static time-kill curves, and showed that polymyxin B and minocycline had a synergistic or additive effect on pan-drug-resistant *Acinetobacter baumannii*, with FICI values ≤0.5 (44%) or in the range of 0.5–1 (48%). Another study conducted animal experiments and observed the four-day survival rate of mice. Polymyxin B and minocycline combination extended the survival time in a neutropenic murine pneumonia model [18]. Maya et al. reported a triple therapy combination against CRAB using an in vitro PK/PD model [24] which demonstrated that minocycline (700 mg loading dose plus 350 mg q12h) and polymyxin B (0.25 mg/kg) combined with sulbactam (9 g, continuous infusion in 24 h) could maintain a bactericidal effect for 96 h with no bacterial regrowth and minimal resistance development [24].

All three strains selected were intermediated to polymyxin B, and two of them were susceptible to minocycline, with one intermediate to minocycline. The MICs of polymyxin B were equal to the MIC_90_ (0.5 mg/L) of 262 *Acinetobacter baumannii* strains from more than 30 teaching hospitals in China [25]. The susceptibility range of minocycline was 20–62.5% against *Acinetobacter baumannii* in Chinese bloodstream infection patients [26]. Hence, all the three strains were typically representative for *Acinetobacter baumannii*. At the same time, the selected strains were classified as ST195, ST208, and ST191, respectively, which were the typical sequence type isolated from sputum, blood, or urine in a previous study [21]. Meanwhile, the three strains all have bla_OXA-23_, which has been considered as the most possible reason for carbapenem resistance in *Acinetobacter baumannii* isolates [21]. For AB070311 and AB170428, Bian et al. [27] performed similar in vitro PK/PD experiments using colistin and sulbactam: 1 mg/L of colistin and 1 g of sulbactam effectively reduced bacterial counts by 2 log_10_CFU/mL in 24 h. We simulated the pharmacokinetics of polymyxin B and minocycline and investigated the antibacterial effect of the combination regimen. Satisfactory results (0.81–1.86 log_10_CFU/mL decrease at 24 h) showed synergy or partial synergy of 0.75 mg/kg polymyxin B and 100 mg minocycline combination against three CRABs in our study. Therefore, polymyxin B combined with minocycline can also be considered as an alternative effective combination approach for treating CRAB. As for PK/PD calculations, MICs in combination are considered more reasonable than MICs in monotherapy. When two antimicrobial agents were used in combination, they had a combined PK/PD index [28]. The tested three strains all achieved the PK/PD target of 2 log_10_CFU/mL reduction (>17.6) for polymyxin B in monotherapy [7]. Unfortunately, none of them reached the PK/PD target 23.3 ± 3.7 for minocycline in monotherapy. In combination, not only the AUC/MICs were achieved for polymyxin B, but also AB070311 and AB170428 reached the AUC/MIC target for minocycline.

Although pharmacokinetic parameters of minocycline were obtained from Chinese healthy subjects [29], there were similar pharmacokinetics between patients and healthy subjects [30,31,32]. Additionally, both polymyxin B and minocycline had similar T_1/2_ (~12 h) in adult patients [30,33], providing more advantageous combination therapy with similar pharmacokinetic profiles. The extended infusion time was taken into consideration in the present study, and a 2 h infusion rather than a single injection was performed. The pharmacokinetic simulations of both drugs were based on two-compartment models with parameters of V_1_, K_21_, α, and β, so the simulated concentrations were more accurate. Our study demonstrated that a combined infusion of 100 mg minocycline and 0.75 mg/kg polymyxin B via a 2 h infusion in humans could lead to the reduction of bacterial burden over 24 h against three CRAB isolates. However, there were several limitations. Firstly, a single dose was performed, and the bactericidal effect of multiple doses needs further validation. Secondly, our study simulated a combination regimen that both drugs were infused simultaneously rather than sequentially, while both approaches could apply in clinics. Whether sequential infusion could also achieve bacterial burden reduction also needs further investigation. Lastly, the host immune system was not taken into account; there could be an underestimation in the extrapolation of our results to patients with a normal immune system. Therefore, the enhanced bactericidal effects of polymyxin B combined with minocycline in the in vitro PK/PD model still needs to be validated in the clinic.

Possible mechanisms could be explained for the synergy between the combination of polymyxin and minocycline. Firstly, polymyxin B is able to destroy bacterial cell envelope [34], which makes it easier for minocycline to enter into cells and bind to the 30S ribosomal subunit [35], thus inhibiting protein synthesis. Secondly, polymyxin B can interfere with the minocycline efflux pump, resulting in increased intracellular concentrations of minocycline and bactericidal activity [18]. Furthermore, the possible PAE may play an important role in this combination. The PAE of tetracyclines was >4 h against Enterobacteriaceae, although unclear against *Acinetobacter baumannii* [36,37,38].

Other than the synergistic effect of the polymyxin B and minocycline, the combination may have other advantages. This combination therapy could prolong the survival time of lung-infection mice [18]. Nephrotoxicity and neurotoxicity are the main limitation of polymyxin B application [4,39]. The incidence of polymyxin B-induced neurotoxicity-related adverse reactions, including perioral paresthesia, dizziness, and numbness of extremities, is much higher in healthy subjects (>70%) [40]. Minocycline is reported to have strong antioxidant activity through chelating with mitochondrial iron [41], which could inhibit the production of reactive oxygen species (ROS) induced by polymyxins and up-regulate the activities of superoxide dismutase (SOD) and catalase (CAT) to enhance antioxidant capacity of nerve cells [42], thus reducing the polymyxins-induced neurotoxicity. Hence, minocycline was tested for a polymyxin-based combination not only because its antibacterial activity [43], but also because its superiority in alleviating adverse reactions, such as the neurotoxicity of polymyxins [42,44]. Of note, the combination could achieve 0.9–3.5 log_10_CFU/mL with a much lower dose of polymyxin B (1.25–1.5 mg/kg, q12 h [45]), which could be expected to reduce the adverse effect of polymyxin B. However, the safety of the combination in clinics still needs further investigation.

## 4. Materials and Methods

Polymyxin B (USP, Lot No. R046V0, 1 mg polymyxin B contained 0.734 mg polymyxin B1, 0.086 mg B1-Ile, and 0.090 mg polymyxin B2) and minocycline hydrochloride (Meilunbio^®^, Dalian, China, MB1477-S, purity 98.9%) were used in this study. Three clinical strains of CRAB isolated from sputum, urine, and blood were studied, detailed clinical information has been described previously [27]. Whole-genome sequencing was employed to investigate the resistant genes and MLST type. Briefly, the genomic DNA was extracted by a Genomic DNA Purification Kit (Tiangen, Beijing, China) according to the production protocol. Sequencing was performed on Illumina Hiseq2500 (Illumina, San Diego, CA, USA). A draft genome was assembled using Velvet (Ver 1.0.15), and resistant genes were searched against “CARD, https://card.mcmaster.ca/” database (accessed on 1 October 2021). Multilocus sequence typing (MLST) was performed according to the Oxford scheme. After comparing sequences to the PubMLST database for *Acinetobacter baumannii* (http://pubmlst.org/abaumannii/ accessed on 1 October 2021), each strain was assigned to the appropriate sequence type.

Minimal inhibitory concentrations (MICs) were determined according to the Clinical and Laboratory Standards Institute (CLSI) guidelines and interpreted according to CLSI breakpoints (CLSI M100-S31, 2021). Checkerboard assays were conducted the same way as that reported by Bian et al. [27]. Synergy was determined by FICI, which is calculated as follows: FICI = MIC PMB when combined with MINO/MIC PMB alone + MIC MINO when combined with PMB/MIC MINO alone [46]. FICI ≤ 0.5 was assigned as synergy, 0.5 < FICI ≤ 0.75 as partial synergy, 0.75 < FICI ≤ 1 as additive effect, and >1 indicated indifferent or antagonistic effect [46].

**Figure 3:**
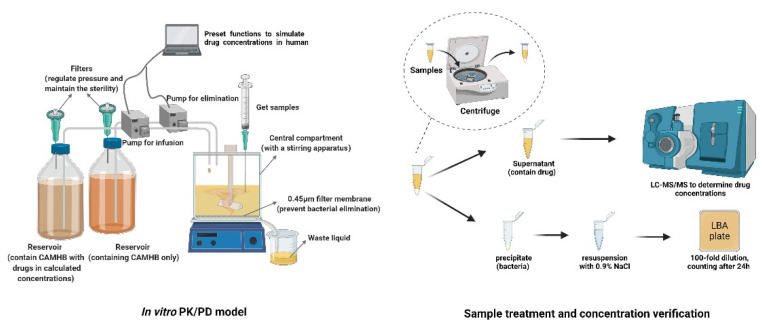
Experimental apparatus and process diagram.

The in vitro PK/PD model was conducted as previously published [27]. Briefly, the volume of Cation-Adjusted Muller–Hinton Broth (CAMHB) medium in the central compartment was maintained at 200 mL. The initial bacterial inoculum was approximately 6 log_10_CFU/mL and was pre-cultured for 1 h before dosing. Two reservoirs containing CAMHB or CAMHB with polymyxin B and minocycline were connected to a peristaltic pump (controlled by WinLIN 3.2 software) and used to simulate drug concentrations in the central compartment (as shown in Figure 3).

The bacterial samples were taken with disposable sterile syringe at 0, 1, 2, 3, 4, 6, 8, 10, 12, and 24 h after the start of infusion for colony counting and concentration validation. The limit of detection for colony counts was 2 log_10_CFU/mL. The dose regimens of single drug or combination of 0.75 mg/kg via a 2 h infusion of polymyxin B, and 100 mg via a 2 h infusion of minocycline were simulated by a two-compartment model, based on the pharmacokinetics from Chinese bloodstream infection patients or healthy volunteers [29,47]. Each regimen for each bacterial strain was performed three times in parallel. Samples for concentration validation were kept in a −80 °C refrigerator until analysis. Both polymyxin B and minocycline concentrations were determined using previously validated liquid chromatography-tandem mass spectrometry (LC-MS/MS) methods [48,49].

For PK/PD calculation, the AUCs of polymyxin B and minocycline were calculated based on the drug concentrations under different dosage regimens. Then, the PK/PD parameters were obtained from AUC of each drug divided by its MIC alone and MIC in combination when FICI reached the minimum.

## 5. Conclusions

Polymyxin B and minocycline combination showed a synergistic or partial synergistic effect against bla_OXA-23_ CRAB strains. The in vitro PK/PD model showed that the dosing regimen of 0.75 mg/kg polymyxin B combined with 100 mg minocycline via a 2 h infusion could be a promising treatment option for CRAB bloodstream infections.

## Figures and Tables

**Table 1:** Genetic information of tested CRAB strains.

ARO Name	AB070311	AB170428	AB162487	Antimicrobial Agents’ Classes
*lpxC*	√	√	√	peptide antibiotic
*lpxA*	√	√	√	
*tetA*	√	√	√	glycylcycline; tetracycline antibiotic
*tetR*	√	√	√	
*adeA*	√	√	√	
*adeB*	√	√	√	
*adeC*	√	√	√	
*adeR*	√	√	√	
*adeS*	√	√	√	
*adeF*	√	√	√	tetracycline antibiotic; fluoroquinolone antibiotic
*adeG*	√	√	√	
*adeH*	√	√	√	
*adeL*	√	√	√	
*adeN*	√			tetracycline antibiotic; fluoroquinolone antibiotic; cephalosporin; macrolide antibiotic; carbapenem; rifamycin antibiotic; lincosamide antibiotic; diaminopyrimidine antibiotic; phenicol antibiotic; penem
*adeI*	√	√	√	
*adeJ*	√	√	√	
*adeK*	√	√	√	
*MexK*	√	√	√	tetracycline antibiotic; triclosan; macrolide antibiotic
*rpsJ*	√	√	√	tetracycline antibiotic
*OXA-66*	√	√	√	cephalosporin; penam
*OXA-366*		√		
*OXA-23*	√	√	√	
*TEM-1*		√	√	cephalosporin; penam; penem; monobactam
*Acinetobacter baumannii OprD conferring resistance to imipenem*	√	√	√	cephalosporin; penam; penem; monobactam; carbapenem; cephamycin
*carO*	√	√	√	carbapenem
*ADC-61*		√	√	cephalosporin
*ADC-78*	√			
MLST	ST195	ST208	ST191	

ARO, Antibiotic Resistance Ontology; *tet*, tigecycline resistance gene; MLST, multilocus sequence typing.

**Table 2:** MICs and FICI values of tested CRAB strains.

Antimicrobial Agents	AB070311	AB170428	AB162487
polymyxin B	0.5 (I)	0.5 (I)	0.25 (I)
minocycline	4 (S)	4 (S)	8 (I)
doxycycline	64 (R)	64 (R)	64 (R)
meropenem	64 (R)	16 (R)	16 (R)
doripenem	64 (R)	32 (R)	16 (R)
aztreonam *	64	64	64
cefoperazone *	>256	>256	>256
sulbactam *	16	16	16
FICI(PMB/MINO)	0.56	0.56	0.375

CRAB, carbapenem-resistant *Acinetobacter baumannii*; MIC, minimal inhibitory concentration, presented in mg/L; FICI(PMB/MINO), fractional inhibitory concentration index of polymyxin B and minocycline combination; S, susceptibility; I, intermediacy; R: resistance. *: CLSI does not provide susceptibility breakpoints for aztreonam, cefoperazone, and sulbactam.

**Table 3:** PK/PD calculations of tested CRAB strains.

	AB070311		AB170428		AB163560	
	MIC	AUC/MIC	MIC	AUC/MIC	MIC	AUC/MIC
PMB Alone	0.50	38.14	0.50	38.14	0.25	76.28
PMB Combined with MINO	0.25	76.28	0.25	76.28	0.06	305.12
MINO Alone	4.00	2.15	4.00	2.15	8.00	1.08
MINO Combined with PMB	0.25	34.42	0.25	34.42	1.00	8.61

MINO: minocycline; PMB: polymyxin B.

## Data Availability

The data that support the findings of this study are available from the corresponding author upon reasonable request.
